# Supplementary material for: Host Shaping Associated Microbiota in Hydrothermal Vent Snails from the Indian Ocean Ridge
Source: Biology (Basel). 2025 Jul 29;14(8):954. doi: 10.3390/biology14080954 (PMC12383625; doi:10.3390/biology14080954)
Supplement: Supplementary file 1 [file biology-14-00954-s001.zip › biology-3702585-supplementary.pdf]

Supplementary data

Additional file S1

Table S1. Geophysical features of sampling sites where deep-sea snails were collected. *

Species	Vent field	Location	Depth (m)	Sampling date	Fluids geochemistry					
					Salty (‰)	pH	T (℃)	CH_4(nM)	H_2(nM)	H_2S(nM)
Chrysomallon squamiferum -white scaly (WC)	Wocan vent, Carlsberg Ridge	60.52824E 6.361367N	2926	Mar,2017	31.7	-	-	140-230	<3.8	1400-2200
Chrysomallon squamiferum -black scaly (BC)	Longqi vent , Southwest Indian Ridge	49.64951E 37.78375S	2780	Feb,2015	32.0	6.25	15	12680.42	8197.05	-
Gigantopelta aegis (G)	Longqi vent , Southwest Indian Ridge	49.64951E 37.78375S	2780	Feb,2015	32.0	6.25	15	12680.42	8197.05	-

*According to the Report of Chinese Cruise DY35 and DY38; -, not determined.

Table S2. Summary of the specimens processed in this study for 16S rRNA amplicon sequence and metagenomics

Species	Specimens	16S rRNA	Metagenome	MAGS
Chrysomallon squamiferum -white scaly (WC1; WC2; WC3)	From the scaly-foot	WC1F; WC2F; WC3F	WC3F	WC3F.bin.9 WC3F.bin.10 WC3F.bin.11 WC3F.bin.15 WC3F.bin.17 WC3F.bin.18
	From the gland	WC1G; WC2G; WC3G	WC1G	WC1G.bin.1 WC1G.bin.2
Chrysomallon squamiferum -black scaly (BC1; BC2; BC3)	From the scaly-foot	BC1F ; BC2F ; BC3F	BC1F	BC1F.bin.5
	From the gland	BC1G; BC2G; BC3G	BC1G	BC1G .bin.1
Gigantopelta aegis (G1; G2; G3)	From the foot	G1F G2F G3F	G1F G3F	G1F.bin.1 G1F.bin.2 G3F.bin1
	From the gland	G1G G2G	-	-

Table S3. Symbiont *Sulfurovum* bins compared with free-living *Sulfurovum* isolates

	BC1F.bin.5	WC3F.bin.9	WC3F.bin.15	WC3F.bin.11	*Sulfurovum riftiae* 1812E^T	*Sulfurovum lithotrophicum* 42BK^T	"Candidatus *Sulfurovum sediminum*" AR	*Sulfurovum* sp. NBC37-1	*Sulfurovum* sp. ALBOsym1
Source	Snail Chrysomallon squamiferum, Longqi hydrothermal field, Southwest Indian Ocean				The vent polychaete, Riftia pachyptila, East Pacific Rise	Deep sea hydrothermal sediments (1033m) in Okinawa,Japon	Deep marine sediments (78m) off Svalbard, Arctic	Sulfide mound in the Iheya North field, Japon(1000m)	Snail Alviniconcha boucheti, PACManus Hydrothermal Vent, Pacific
Completeness	82.77	87.89	85.38	89.95	100.00	100.00	Draft genome	100	98.36
Genome size (Mbp)	1.42	1.38	1.61	2.03	2.37	2.22	2.12	2.56	2.26
GC Content (%)	44.77	46.1	38.91	31.71	47.4	44.26	39.4	43.8	37.3
Number of protein coding gene	1478	1438	1640	1906	2317	2217	2248	2466	2346
Accsession number	6666666.654594	6666666.654595	6666666.654596	6666666.654597	GCA_001595645.1	CP011308.1	AJLE01000000	AP009179	VCAW00000000

Additional file S2

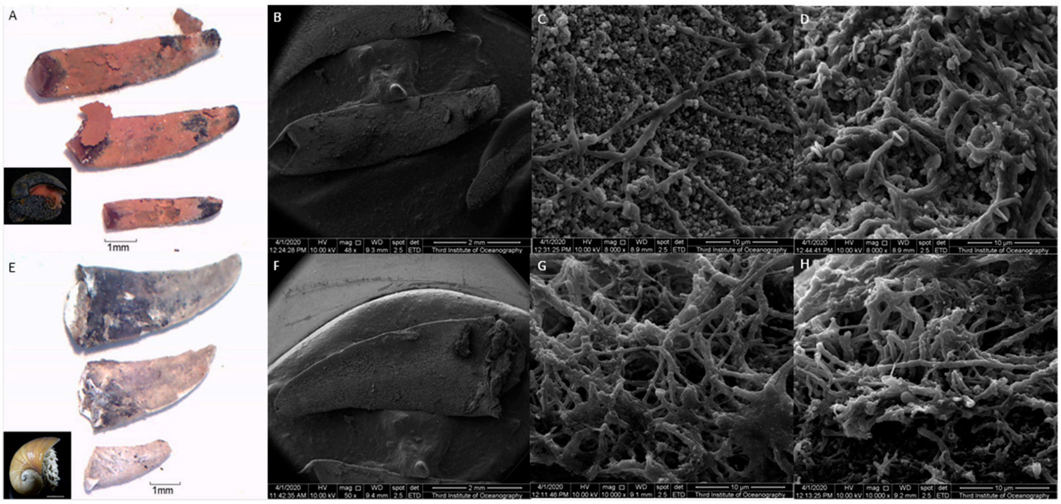

Figure S1. Microscopic and scanning electron micrographs showing sclerites of the scaly snails. Individual sclerites (A, B) of *Chrysomallon squamiferum* (black scaly) covered with heavy iron sulfide mineral and filamentous and coccoid bacteria on the scale surface (C, D); individual sclerites (E, F) of *Chrysomallon squamiferum* (white scaly) covered with less mineral and mainly filamentous bacteria on the scale surface (G, H).

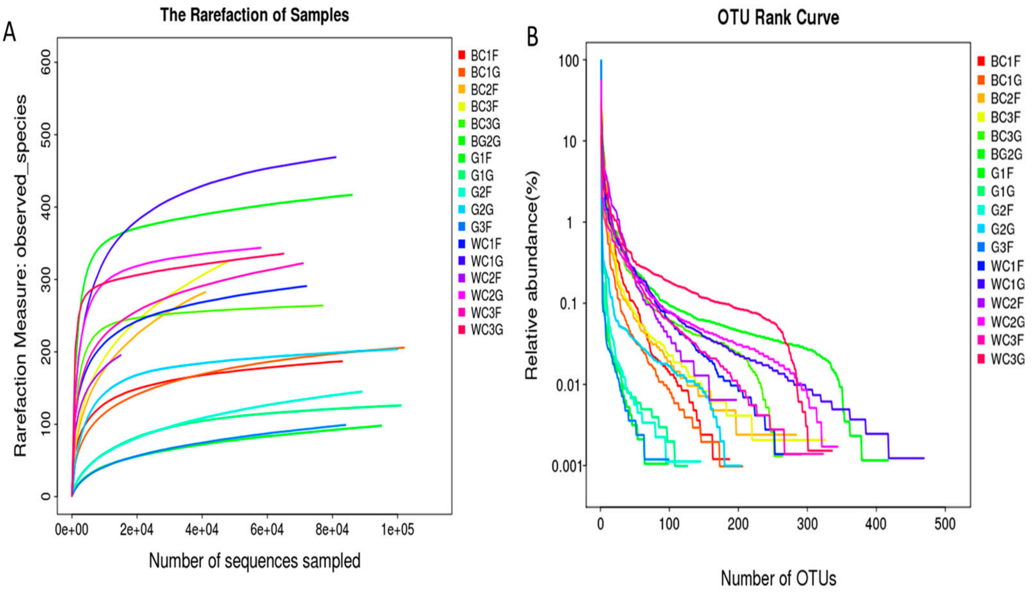

Figure S2. Rarefaction curves (A) and rank abundance curves (B) of each sample. BC, black scaly *Chrysomallon squamiferum*; WC, white scaly *Chrysomallon squamiferum*;

G, *Gigantopelta aegis.* A. Rarefaction curves on each sample constructed by using observed ASVs; B. Rank–abundance curves. The abscissa represents the ranking level of the ASV number, and the ordinate represents the relative percentage of the ASV number.

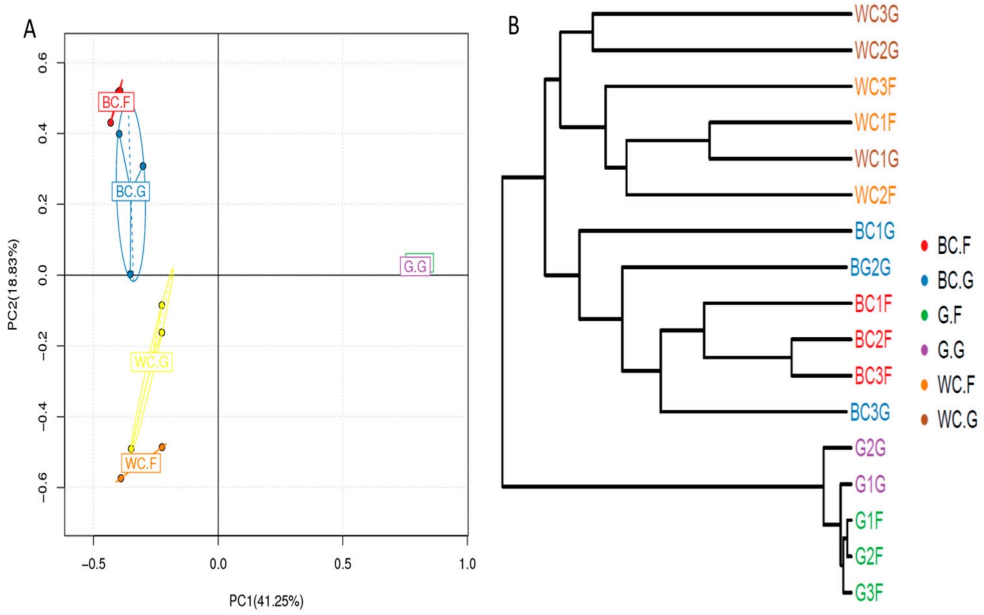

Figure S3. PCoA analysis (A) and the Bray–Curtis cluster tree (B), showing results of beta diversity analysis of hydrothermal snails. BC, black scaly *Chrysomallon squamiferum*; WC, white scaly *Chrysomallon squamiferum*; G, *Gigantopelta aegis*; F, scaly-foot or foot; G, glands

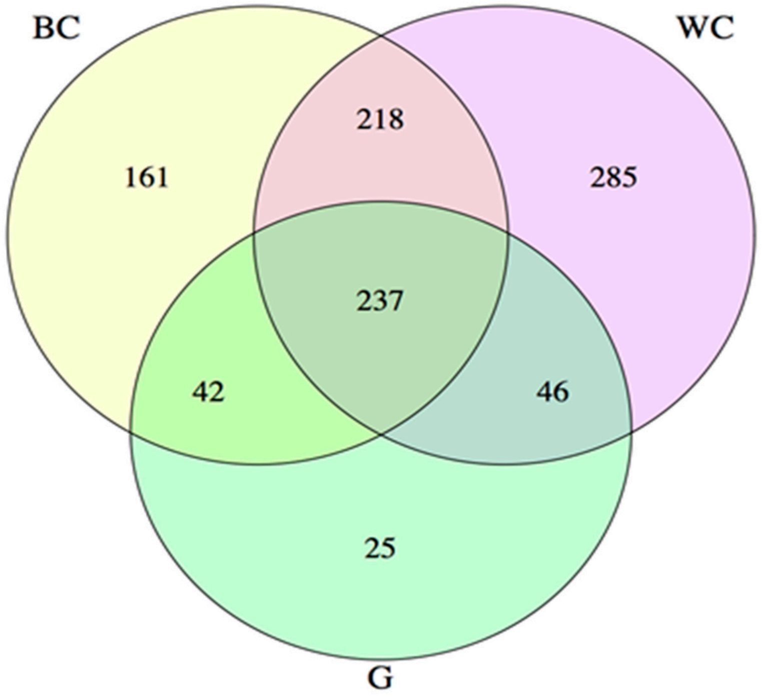

Figure S4. Venn diagram describing the ASV distribution among three snails. BC, black scaly *Chrysomallon squamiferum*; WC, white scaly *Chrysomallon squamiferum*; G, *Gigantopelta aegis*.

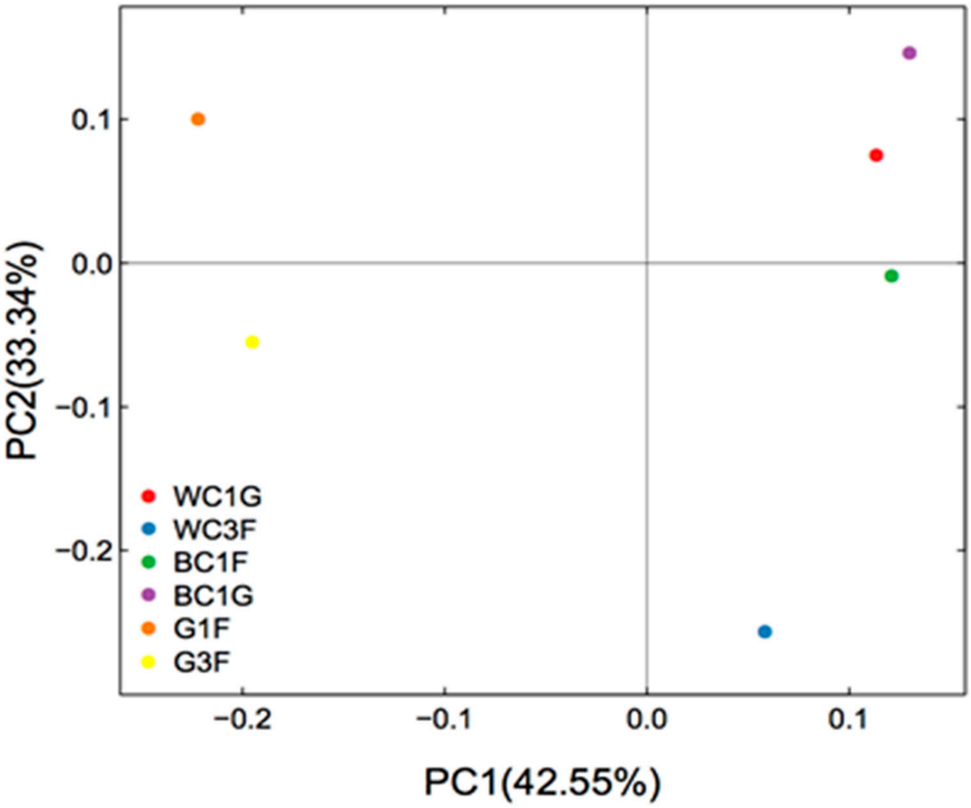

Figure S5. PCA analysis results based on functional abundance of level 3 KEGG pathways.

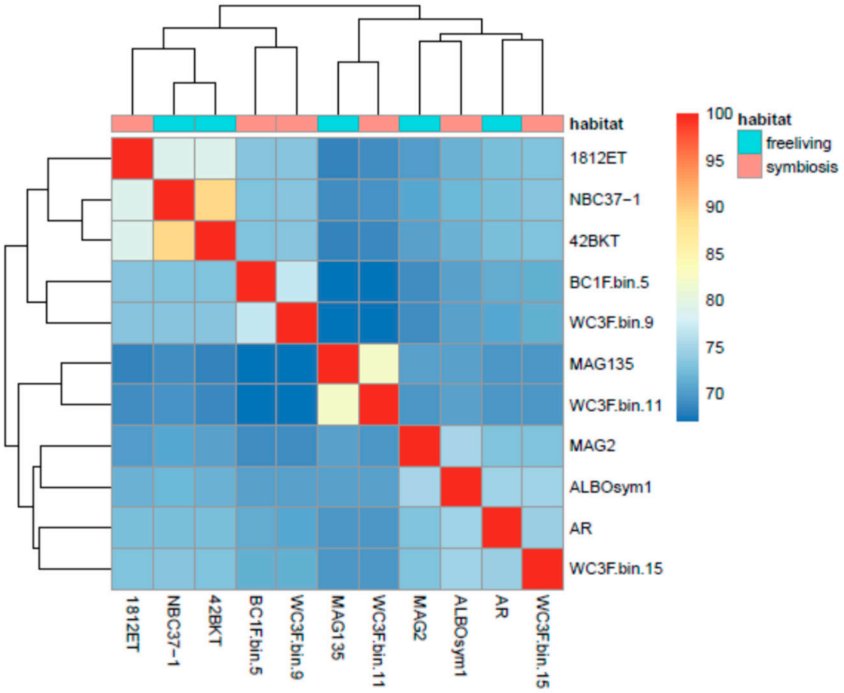

Figure S6. Heatmap of pairwise amino acid identity (ANI) values and phylogenomic tree in the genus *Sulfurvum.*

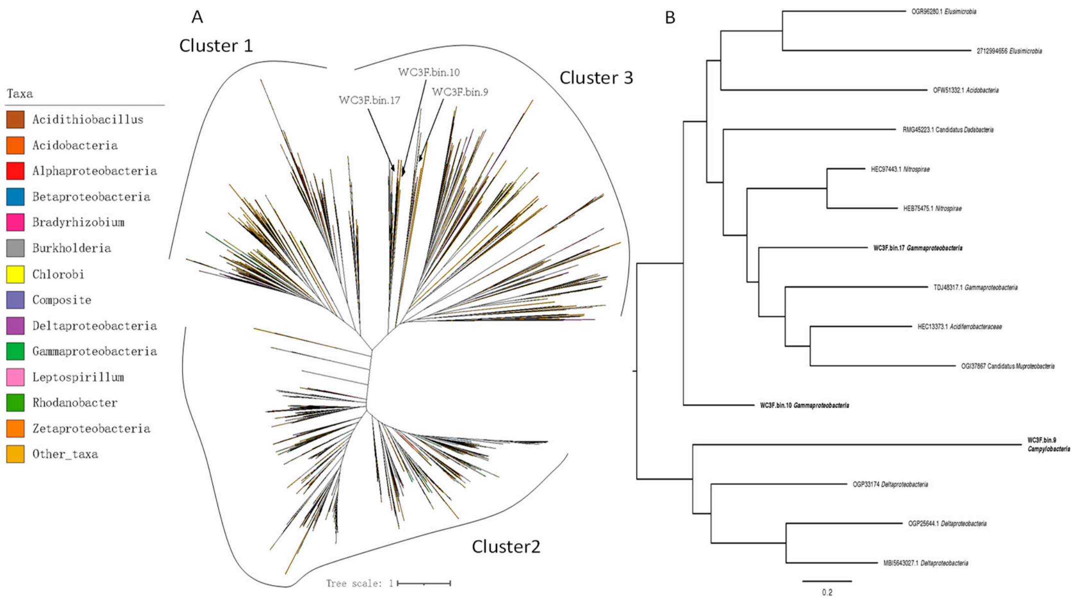

Figure S7. Cyc2 maximum likelihood phylogenetic tree (300 bootstraps) with all (A) and the close neighbor (B) sequences showing the relative phylogenetic position of Cyc2 belonging to three MAGs identified in this study.
